# Intervertebral disc calcification in a child

**DOI:** 10.4103/0019-5413.43401

**Published:** 2008

**Authors:** Athar M Ahemad, Bibhas Dasgupta, Jairam Jagiasi

**Affiliations:** Department of Orthopedics, Sion Hospital, Mumbai - 400 022, India

**Keywords:** Intervertebral disc calcification, neck pain, torticollis

## Abstract

Disc calcification in children is a rare condition of which only approximately 200 cases have been reported worldwide and one from India and we report one such case. A five year–old boy presented with neck pain, torticollis and limitations of cervical motions following a fall while playing 3 months back. He had low grade fever cervical lymphadenopthy, paraspinal muscle spasm. His blood counts and ESR was raised. Fine needle aspiration cytology of lymph node revealed reactive lymphadenitis. His cervical radiograph slowed calcification of C 6-7. MRI scan showed hypointense signals in C6-C7 and D5-D6 disc on both T1 and T2 W images. Cerebrospinal fluid examination was normal. He improved on analgesics, bed rest and cervical traction.

Calcification of intervertebral disc is a rare condition in children which presents itself with symptoms of pain, limitation of motion of spine, torticollis with fever, raised white blood cell count and elevated erythrocyte sedimentation rate in some. It can also occur as an incidental finding on radiographs.^1^ The exact cause is not known but precedent trauma and upper respiratory infection are most commonly seen. A distinction has been made between symptomatic and asymptomatic disc calcification, with the former being mostly cervical in distribution.^2^ The clinical course is self-limited and a conservative therapy (rest, analgesics, cervical traction or collar) is usually effective.^2^–^4^ Here, we describe a case of two-level disc calcification in a five-year-old boy presenting with neck pain and stiffness. Our first clinical impression was of cervical spine tuberculosis, which is expected, considering the high prevalence of spinal tuberculosis in India.

## CASE REPORT

**Figure 1:**
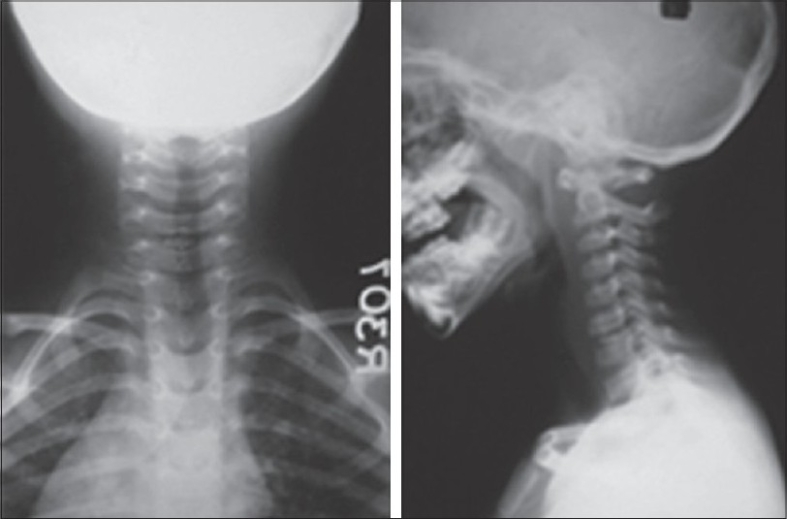
Cervical spine radiographs shows calcified disc at C6-C7 level

**Figure 2:**
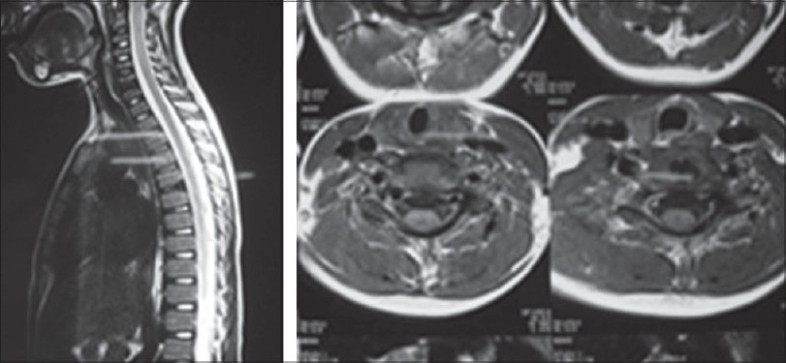
MR images showing hypointense signals at the levels of calcified discs

A five-year-old boy came to us with a history of trauma to the neck due to fall while playing three months back leading to neck pain, torticollis and limitation of cervical motion. The pain was relieved to some extent after 10-15 days by massage at home and analgesics given by a local practitioner. He had high-grade fever at the time of the fall which subsided over few days presumably with antipyretics. On admission, he complained of neck pain and stiffness, abnormal deviation of neck and intermittent low-grade fever. He had right-sided torticollis with paraspinal muscle spasm with restriction of lateral flexion of neck to opposite side and cervical lymphadenopathy on same side. There was no evidence of any neurological deficit. Total leukocyte count was 12000 with a normal differential and erythrocyte sedimentation rate of 100 mm. The FNAC of the cervical nodes revealed reactive lymphadenitis. Cervical radiographs showed calcification of C6-C7 disc space [Figure 1]. The CSF puncture revealed normal findings ruling out meningitis. An MRI scan done showed hypointense signals in C6-C7 and D5-D6 discs on both T1 and T2 weighted images suggestive of calcification and no cord or root compression [Figure 2]. During a fortnight of hospitalization, symptoms diminished with no other treatment than bed rest, analgesics and cervical traction. Then he was put on cervical collar and neck was gradually mobilized with active exercises.

## DISCUSSION

The etiology of pediatric disc calcification remains unclear.^3^ Antecedent trauma has been blamed but the relationship is weakened by the fact that disc calcification has been reported in patients with no history of trauma and even in newborns.^5^ Some authors have speculated on an inflammatory or infectious mechanism, as evidenced by fever and raised counts. However, these children recover without any antibiotic therapy.

A high index of suspicion and awareness of the condition among clinicians is a must for the diagnosis of disc calcification. It needs to be differentiated from the much commoner cervical spine tuberculosis in the Indian setup. Radiographs of the cervical spine are sufficient for the diagnosis in most cases. An MRI is advisable only in cases with a neurological loss. In febrile cases the condition can be mistaken for meningitis. In the present case, the child has responded well to a conservative regime without any antibiotic therapy despite the fact that he initially had fever and elevated counts. This makes an infective etiology very unlikely and leaves only trauma as the probable cause. Yet, no factor has been found to be conclusively related to the natural history of the disease in the literature.^3^
